# The Blessed Union of Glycobiology and Immunology: A Marriage That Worked

**DOI:** 10.3390/medicines10020015

**Published:** 2023-01-19

**Authors:** Jhenifer Santos dos Reis, Israel Diniz-Lima, Marcos André Rodrigues da Costa Santos, Pedro Marçal Barcelos, Kelli Monteiro da Costa, Raphael do Carmo Valente, Lorrane de Souza Chaves, Luma Petel de Campos, Ariely Costa dos Santos, Rafaela Gomes Correia de Lima, Debora Decote-Ricardo, Alexandre Morrot, Jose Osvaldo Previato, Lucia Mendonça-Previato, Celio Geraldo Freire-de-Lima, Leonardo Marques da Fonseca, Leonardo Freire-de-Lima

**Affiliations:** 1 Instituto de Biofisica Carlos Chagas Filho, Universidade Federal do Rio de Janeiro, Rio de Janeiro 21941-902, Brazil; 2 Núcleo Multidisciplinar de Pesquisa em Biologia, Universidade Federal do Rio de Janeiro, Campus Duque de Caxias, Rio de Janeiro 25250-470, Brazil; 3 Instituto de Veterinária, Departamento de Microbiologia e Imunologia Veterinária, Universidade Federal, Rio de Janeiro 23890-000, Brazil; 4 Instituto Oswaldo Cruz, Laboratório de Imunoparasitologia, Rio de Janeiro 21040-360, Brazil; 5 Faculdade de Medicina, Universidade Federal do Rio de Janeiro, Rio de Janeiro 21941-902, Brazil

**Keywords:** glycoimmunology, glycoconjugates, lectins, galectins, siglecs, oncofetal antigens

## Abstract

In this article, we discuss the main aspects regarding the recognition of cell surface glycoconjugates and the immunomodulation of responses against the progression of certain pathologies, such as cancer and infectious diseases. In the first part, we talk about different aspects of glycoconjugates and delve deeper into the importance of N-glycans in cancer immunotherapy. Then, we describe two important lectin families that have been very well studied in the last 20 years. Examples include the sialic acid-binding immunoglobulin (Ig)-like lectins (siglecs), and galectins. Finally, we discuss a topic that needs to be better addressed in the field of glycoimmunology: the impact of oncofetal antigens on the cells of the immune system. New findings in this area are of great importance for advancement, especially in the field of oncology, since it is already known that cellular interactions mediated by carbohydrate–carbohydrate and/or carbohydrate proteins are able to modulate the progression of different types of cancer in events that compromise the functionality of the immune responses.

## 1. Introduction

The fields of cancer glycobiology and glycobiology of infectious diseases provide crucial information concerning the cell surface glycoconjugates, as they play an important role in immunosurveillance during the development and establishment of certain pathologies [1]. Furthermore, screening for atypical glycophenotypes culminates in the construction and modulation of an innate and adaptive immune response, mainly because glycans are biological structures that are very well conserved by evolution and are naturally heterogeneous, and end up acting as carriers of biological information that are decoded by families of proteins known as lectins [2,3].

The effects of the structural recognition of glycans by these receptors, present mainly in cells of the immune system, are paramount in defining the immune responses. Therefore, those receptors are subjected to subversion of the host response against certain pathologies, being involved in the persistence of infections and tumors resistant to chemotherapy and increased metastatic potential [4]. Bearing in mind the great complexity of these themes, in this review we sought to unfold the relationships between glycoconjugates and the host’s immune response, both in aspects favorable to the pathological progression, and in the modulation of the immune response.

## 2. Cell Surface Glycoconjugates: A Hallmark of All Living Cells

For the last 30 years, it has been well accepted that glycans cover the cell surface of all living cells [5,6]. The first glycoprotein identified in eukaryotes was described over 80 years ago [7]. At that time, it was not thought that cell surface glycoconjugates could influence the behavior of different cell types. Further studies have confirmed that this important type of posttranslational modification (PTM), which is named glycosylation, is not restricted to higher organisms [6]. Glycan-carrying proteins have also been found in parasitic protozoa, virus, fungi and prokaryotes, such as bacteria and archaebacteria [8,9,10,11,12].

Although the expression of glycoproteins is a common feature among different organisms, glycans and/or monosaccharide structures are differentially expressed among them. A clear example are the mammalian cells, which express glycans much more diversely than other organisms [13]. Considering that the glycans carried by glycoproteins are fundamental for life, it would be plausible to propose that such differences may have played an important role in speciation, and in the formation of different organs and tissues in multicellular organisms [14]. This explanation highlights the importance of understanding glycan biology in human health and disease, mainly because there is also a diversified expression of receptors with affinity to specific glycoconjugates, such as the differentiated expression of types of galectins in different tissues of the organism [15]. Furthermore, during development, in the phases of mammalian fetal life, there is differential expression of glycoconjugates in relation to normal adult tissues [16]. In mammalian cells, during glycoprotein biosynthesis, these molecules may be transferred from the endoplasmic reticulum to the Golgi apparatus, and finally transported to the cell membrane [13], where they are capable of influencing the behavior of different cell types, including cells of the immune system [17,18].

As it is an emerging field within immunology and remains a very little commented subject in the classrooms of different biomedical courses [19], before addressing the impact of glycan structures carried by proteins in cells of the immune system, it is important to emphasize the concept of glycosylation, which is mediated by a harmonized set of enzymes, named glycosyltransferases and glycosidases [20]. While glycosyltransferases are responsible for the transfer of a sugar from a nucleotide sugar donor to a substrate, the glycosidases catalyze the hydrolysis of glycosidic bonds in glycan structures [21]. Today, it is well known that genes encoding this glycosylation machinery represent over 1% of the total genome [22,23]. These enzymes are expressed in a finely regulated way, which depends on cell activation, metabolic status and microenvironmental features [24].

The addition or removal of sugars in glycans that decorate polypeptide chains generate numerous structural variations to a given protein, favoring the emergence of identical polypeptide chains decorated with different glycan structures, which today we know as glycoforms [25,26]. The multitude of glycans and enzymes involved in their biosynthesis gives the mammalian glycome a huge potential of glycan structures, which expands the diversity already created by the proteome [13,22,23]. Nowadays, within the mammalian glycosylation repertoire, *N*- and *O*-glycosylation are among the most studied PTM [13]. In a protein backbone, the existence of potential *N*- and *O*-linked glycosylation sites, together with the presence or absence of glycosidases and glycosyltransferases are crucial characteristics in determining the degree of the glycosylation of a given protein [26].

## 3. *N*-Glycans in Cancer Immunotherapy

**Figure 1:**
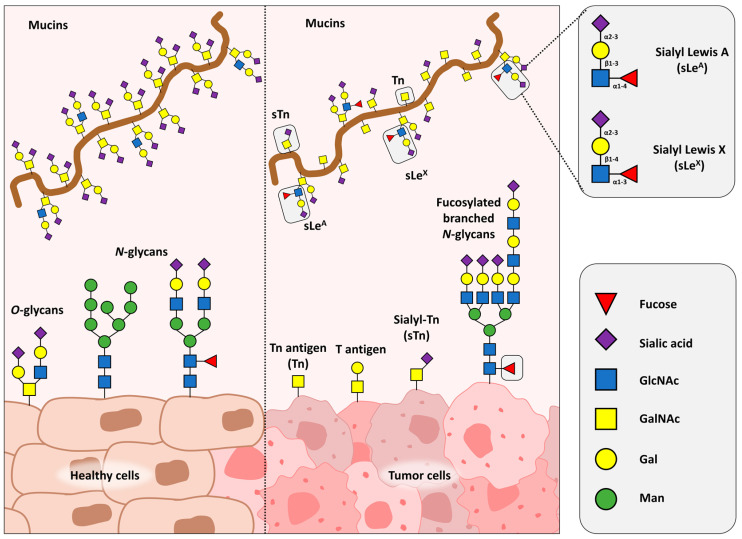
Glycosylation changes in cancer cells compared to healthy tissues. Normal pattern of glycosylations are shown in the left panel, whereas the right is associated with cancer cells. Changes in O- and N-linked glycan structures are displayed, as well as the differences between Sialyl Lewis A and Sialyl Lewis X.

Cancer cells present an altered repertoire of glycoconjugates and this aberrant glycosylation pattern has been established as a cancer hallmark [27] (Figure 1). Regarding *N*-glycans, the *β*1,6-GlcNAc–branched *N*-glycans are widely overexpressed in cancer cells, being associated with increased expression of *N*-acetylglucosaminyltransferase V (GnT-V), responsible for its biosynthesis, which is encoded by the MGAT5 gene [28,29]. Immune evasion is another one of the cancer hallmarks, occurring through varied mechanisms, such as downregulation of MHC class I [30] and T cell death induction [31]. Several glycoconjugates have been associated with protection of tumor cells against the immune system attack [32].

In a remarkable and well-designed work, Silva and colleagues observed that human samples from colorectal cancer presented high expression of *β*1,6-GlcNAc-branched *N*-glycans and Gnt-V enzyme. An increase in the differentiation or recruitment of Foxp3^+^ Tregs was also observed, which is associated with immunosuppression in the tumor microenvironment. Furthermore, the coculture of MKN45 T5, cells that overexpress MGAT5, and PBMCs demonstrated that the increase in the biosynthesis of branched *N*-linked glycans led to the internalization of MHC-I, reduced release of the proinflammatory cytokines IL-6 and IL-8, and increased release of inhibitory cytokine TGF-β. This was associated with masking of immunogenic glycan mannose epitopes which are recognized by antigen-presenting cells (APC), such as DCs that express glycan-recognizing receptors, namely DC-SIGN and MR. On the other hand, compromising the appearance of atypical *N*-glycan structures on the surface of tumor cells, either by using inhibitors or by knocking out the MGAT5 gene, led to an increase in release of proinflammatory cytokines and in the antitumor immune response. These findings show the importance of *N*-glycosylation in modulation antitumor immune response and, therefore, cancer immunotherapy [33].

*β*1,6-GlcNAc–branched *N*-glycans and MGAT5 also present an essential role in regulation of the immune system, since it has been widely reported that mice deficient in MGAT5, and therefore β1,6-GlcNAc–branched *N*-glycans, are highly susceptible to autoimmune diseases [34,35]. Furthermore, branched *N*-glycans also present a central role in T cell biology targeting different T cell receptors (such as TCR, CD25, and CD4), thereby regulating T cell proliferation, T cell differentiation, T cell signaling and the production of inflammatory cytokines [36]. Activation of T cells via T cell receptors (TCR) promotes the upregulation of the MGAT5 gene, which in turn leads to GnT-V–mediated glycosylation of the TCR [37]. This creates a ligand for galectin 3, which is responsible for holding CD45 and the TCR signaling complex in close proximity via their glycans forming a molecular lattice [38]. Consequently, CD45 phosphatase activity induces downregulation of T cell signaling, preventing low-avidity T cell activation [34]. TCR activation also leads to in increased *N*-glycan branching on CTLA-4, which elevates its retention on the T cell surface, suppressing T cell activation [39] and promoting Th2 development over Th1 responses [40].

One mechanism of immune evasion that has been explored in recent years as a target for cancer immunotherapy is the PD-1–PD-L1 pathway. Programmed cell death 1 (PD-1) is present on the surface of B-cells, T-cells, natural killer (NK) cells, dendritic cells, monocytes, and tumor-infiltrating lymphocytes (TILs), while PD-L1 is expressed in cancer cells and APC [41,42]. PD-L1 disrupts intracellular signaling and downregulation of effector T cell function, acting therefore as an immune checkpoint that mediates coinhibitory signals to T cell activation [41]. Cancer cells overexpress PD-1 due to activation of several signaling pathways that are crucial to tumorigenesis. This leads to inhibition of T cell activation, proliferation, and survival and cytotoxic secretion within cancer cells, which promotes induction and maintenance of immune tolerance within the tumor microenvironment [43]. The therapeutic potential of targeting the PD-1–PD-L1 axis has been evidenced by the Nobel Prize of 2018, which promoted the approval of immunotherapy targeting this pathway in several solid tumors [44]. However, anti-PD-1–PD-L1 therapy has been facing several obstacles. First, the application of this therapy relies on the detection of PD-L1 in cancer cells. Second, a great number of patients present primary or acquired resistance to PD-1–PD-L1 blockade [45]. Despite the mechanisms of resistance not being fully understood, it has been demonstrated that *N*-glycosylation plays an important role in this pathway.

Recent studies have shown that PD-L1 is highly *N*- glycosylated in the majority of cancer cells in which it is expressed [46,47]. Li and colleagues showed that PD-L1 N192, N200 and N219 glycosylation induces its stabilization, while nonglycosylated PD-1 is phosphorylated by glycogen synthase kinase 3β (GSK3β), which induces its phosphorylation-dependent proteasome degradation [48]. Furthermore, in following work Hung described that this glycosylation is essential for the interaction between PD-L1–PD-1 and targeting glycosylated PD-L1 (gPD-L1) blocks PD-L1–PD-1 interaction and promotes PD-L1 internalization and degradation [49]. *N*-glycosylation of PD-L1 is not only important for its function but also its detection [50]. On the other hand, Liu and colleagues described that PD-1 N58 glycosylation promotes the interaction with camrelizumab, a recently approved PD-1-specific monoclonal antibody, while the efficiency of camrelizumab to inhibit the binding of PD-1 is substantially reduced for glycosylation-deficient PD-1 [51]. Lu et al also showed that PD-1 N58 glycosylation is essential to binding and blocking efficacy of cemiplimab, another monoclonal antibody approved in 2018 [52]. These findings evidence that the glycosylation status of PD-L1 and PD-1 directly impact immunotherapy response and therefore should be taking into consideration while developing anti-PD-1–PD-L1 strategies.

## 4. Lectins as Decoders of Biological Information in Cellular Glycoconjugates

Many published papers, especially in the oncology field, have demonstrated that both unusual glycan structures and lectins (proteins that have a carbohydrate-recognizing domain) expressed by both tumor stroma and transformed cells are able to modulate cancer development and progression [53,54]. Among the most classic and well-studied examples is the role mediated by the tetrasaccharide Sialyl-Lewis X (sLeX), which serves as a ligand for the set of cell adhesion proteins known as selectins. This interaction allows adhesion of cancer cells and leukocytes to endothelial cells within capillaries, supporting their extravasation into tissues [55].

It is well accepted that lectins have a central role in cell biology, since they are able to translate glycan-encoded information into bioactivity [56]. Among the most studied lectins, galectins and siglecs stand out. While galectins present high affinity for linear polylactosamine chains [57,58], siglecs bind exclusively to sialic acid (Sia)-containing glycoconjugates [59,60].

### 4.1. Lectins as Tools for N- and O-Glycan Detection and Purification

The capacity of lectins to recognize and bind to specific glycan chains has been historically explored as a tool for the separation and detection of glycans in different analytical techniques. Certain groups of lectins have an affinity for *N*-linked, *O*-linked glycoproteins or both types (Table 1) and since they are not species-specific, their spectrum of application is wider than that of antibodies [61].

Lectins are widely used in histochemistry and cytochemistry to detect glycoconjugates in cells and tissues [62]. One way of visualizing lectin-binding sites is an indirect method employing lectins conjugated to a hapten, such as digoxigenin, which is then recognized using enzyme-linked streptavidin [63]. Lectin blotting or lectin-probed Western is a variation of the traditional Western blot, in which lectins are also employed to detect glycoproteins [64]. The lectin blot is very similar to the traditional Western blot, the main difference being that the membrane is then incubated with a specific lectin and labeled with a group, such as digoxigenin (DIG) that will further bind to a secondary antibody conjugated to an enzyme that catalyzes a color-producing reaction (alkaline phosphatase) or a more sensitive luminescence-producing reaction (horseradish peroxidase) [65]. Fluorochrome-labeled lectins can also be used to detect glycans on the cell surface of live cells by flow cytometer [66]. To purify glycoconjugates, lectin affinity chromatography can be applied (LAC). LAC utilizes different immobilized lectins that bind glycoproteins noncovalently and reversibly, and therefore they may be selectively released from an affinity column by competitive elution using a specific corresponding free sugar or sugar analog [67].

Lectin microarrays, which were developed in 2005 [68], are used for characterizing glycosylation profiles in diverse clinical situations, especially in cancer biomarker discovery [69,70]. In this technique, lectins are immobilized on a solid surface, and binding of target glycoproteins can be detected either directly through their prior labeling with fluorescent reagents, or indirectly by overlaying a fluorescently labeled relevant antibody raised against the target glycoprotein (or via biotinylated antibody and fluorescently labeled streptavidin). The microarray is scanned, followed by the interpretation of the signals [71,72], and structural information about the glycome is obtained using the known glycan-binding specificities of the lectins [71]. The utilization of multiple lectins in the microarray allows the studying of multiple lectin–glycan interactions in a single experiment and holds the promise of enabling glycomic profiling of cancers in a fast and efficient manner [73]. However, the sensitivity, simplicity, and robustness of lectin microarrays require further improvement to broaden their application [69].

**Table 1:** Common lectins used for detection and purification of *N*- and *O*-linked glycans and their main specificity.

Lectin	Main Specificity	Reference
*Vicia villosa* (VVL)	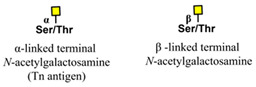	[74]
*Helix pomatia agglutinin* (HPA)	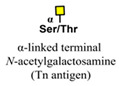	[75]
*Peanut agglutinin* (PNA)	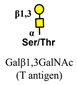	[76]
*Sambucus nigra* (SNA)	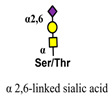	[77]
*Maackia amurensis* (MAA)	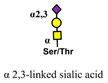	[78]
*Concanavalin A* (ConA)	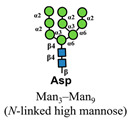	[79]
*Phaseolus vulgaris-E* (PHA-E)	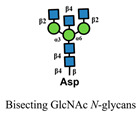	[80]
*Phaseolus vulgaris-L* (PHA-L)	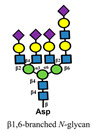	[81]

### 4.2. Galectins

Regarding galectins, numerous works have demonstrated this family of lectins is able to influence many events related to assembly of the immune response [82,83,84,85]. Over 15 years ago, outstanding papers published by Rabinovich’s and Baum’s groups were essential to foster the emergence of the glycoimmunology field. Many articles confirmed that galectins, especially galectin-1 (Gal-1), are able to induce the maturation [86], proliferation [87,88] and apoptosis of immune cells [89,90], playing an important role in the development and maintenance of a healthy immune system [22,91,92]. On the subject of T cell biology, at the beginning of the 21st century, with the advancement of glycoimmunology, several papers confirmed that different glycan structures modulate T cell-related biological phenomena, such as activation, differentiation, death, and homing, by either generating or masking ligands for endogenous lectins [93,94,95,96]. In 2014, Croci and colleagues identified a glycosylation-dependent pathway involving the participation of Gal-1 that compensates for the absence of cognate ligand and preserves angiogenesis in response to vascular endothelial growth factor (VEGF) blockade. The authors observed that the remodeling of glycans decorating the endothelial cells regulates the binding of Gal-1 to *N*-linked glycans found in the vascular endothelial growth receptor (VEGFR), influencing the efficacy of the anti-VEGF treatment [97]. Besides governing tumor angiogenesis [97,98], Gal-1 is involved in the emergence of CD8^+^CD122^+^PD-1^+^ Treg cells [99], activation of signaling pathways linked to the epithelial–mesenchymal transition (EMT) process [100,101], acquisition of drug resistance phenotype [102], cancer cell proliferation, migration and metastasis [103,104], among others.

Recent studies have demonstrated that Gal-2 plays an important role in the immunological pathomechanism of preeclampsia [105] and its expression is linked to gestational diabetes, which may contribute to the emerging understanding of the role of immunomodulation and inflammation in gestational diabetes mellitus [106]. Gal-3 has also been very well studied, and several papers have demonstrated its participation in the modulation of the immune system in health and pathological conditions. For example, in human and murine atherosclerotic plaques, Gal-3 is vastly expressed by macrophages (Mɸ), promoting a deleterious role on plaque development through augmentation of the inflammatory response [107,108]. Although this has not been fully established, other groups have proposed that Gal-3 presents a supportive effect through modulation of the inflammatory profile mediated by Mɸ, exerting both anti-inflammatory and profibrotic properties [109]. Recent studies demonstrate that high levels of circulating Gal-3 are strictly associated with diabetes and its complications. The increased expression of Gal-3 in pancreatic beta-cells affects both glucose metabolism and glycoregulation in mice on a high-fat diet, disturbing the fasting glycemic values and glycemia [110]. Quenum Zangbede and colleagues demonstrated that Gal-3 in M2-Mɸ regulates neutrophil turnover, displaying a protecting role by solving neuropathological features in brain during parasitic infections [111]. Gal-9 also presents important functions on Mɸ biology, since it is able to regulate M1 vs. M2 polarization in RAW264.7 cells [112]. Gal-4, which is detected only in the digestive tract [113], has been identified as a potential inducer of CD4+ T cells to exacerbate intestinal inflammation [114]. Interestingly, it has been evinced that Gal-4 specifically stimulates CD4+ T cells, but not other immune cells such as Mɸ and B cells to express the cytokine IL-6 [114], a well-known stimulus involved in the pathogenesis of not only intestinal inflammation but also colon cancer [115].

**Figure 2:**
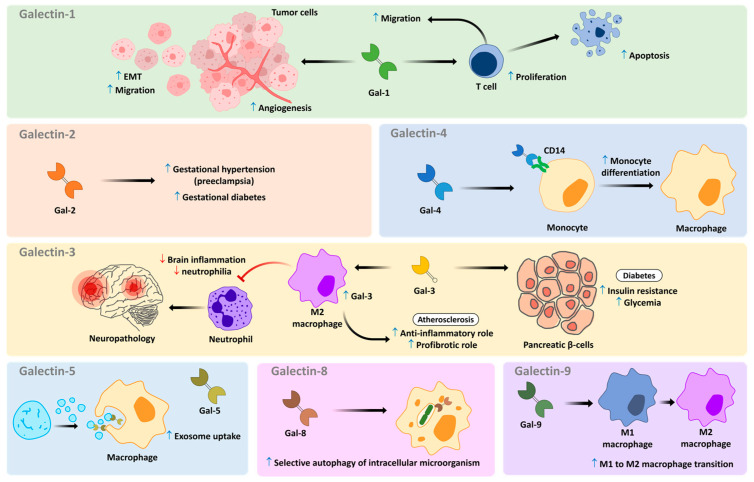
Differential role of galectins under aspects of immune system function and pathological conditions. Small arrows in blue and red indicate upregulation and downregulation, respectively.

Regarding Gal-5, it has been shown to bind to the surface of exosomes secreted by rat reticulocytes, modulating the uptake of vesicles by Mɸ [116]. The differentiation of monocytes to Mɸ may also be modulated by Gal-4, which binds to CD14, triggering the activation of the MAPK signaling pathway [117]. Recently, it was demonstrated that in Mɸ, Gal-8 recognizes damaged *Mycobacterium tuberculosis*-containing phagosomes, and directs the microorganism to selective autophagy, highlighting the importance of Gal-8 in the innate immune response to this pathogenic bacterium [118]. Some of the effects of different galectins in the immune system are summarized in Figure 2.

### 4.3. Siglecs

**Figure 3:**
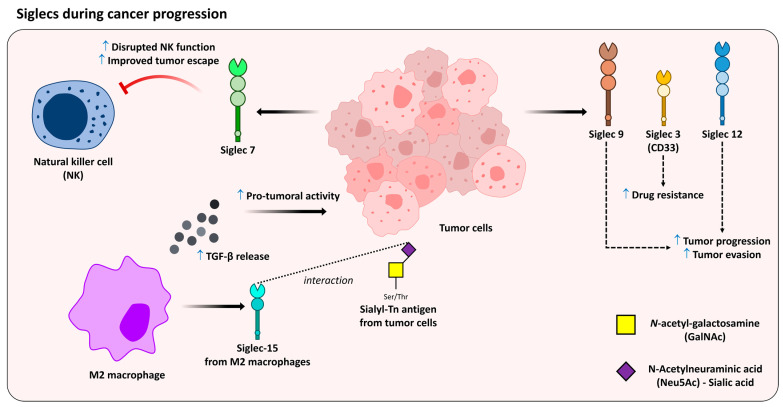
Differential role of siglecs during cancer progression. Siglecs expressed in tumor cells and M2 macrophages contribute to protumorigenic effects. Dashed line indicates receptor interaction and dashed arrows indicate successive effects.

Siglecs are I-type (immunoglobulin superfamily–type) lectins and exert functions in the immune system in events related to cell adhesion, pathogen recognition, cell activation, signaling, and death, among others [119,120,121,122] (Figure 3). Although many glycan-binding proteins (GBPs) can recognize Sia-containing glycans, siglecs show great specificity for them, forming extensive molecular interactions [119,123,124]. It has been well described that the on the cell surface, most living cells, including mammalian cells, are highly decorated with Sia-carrying glycans, which in most situations are able to favor siglec binding to the surface of the same or adjacent cells [125]. This phenomenon may be dynamically modulated in vivo through sialidases, also known as neuraminidases, disrupting cell interactions that occur between Sia-containing glycans [59]. It is also important to point out that most siglecs present immunoreceptor tyrosine-based inhibition motifs, also known as ITIMs, in their cytoplasmic domain, which are able to conduct inhibitory events. In addition, some siglecs also present an immunoreceptor tyrosine-based switch motifs (ITSMs), which can act in inhibitory or activating events [60,126]. A few siglecs act in association with other cell-surface proteins that contain immunoreceptor tyrosine-based activation motifs, resulting in cell activation, such as siglec E, which can modulate dendritic cell activation and potentially influence antigen presentation [127]. In addition, siglec 2 (CD22) has been implicated in B-cell activation in non-Hodgkin lymphoma [128].

**Figure 4:**
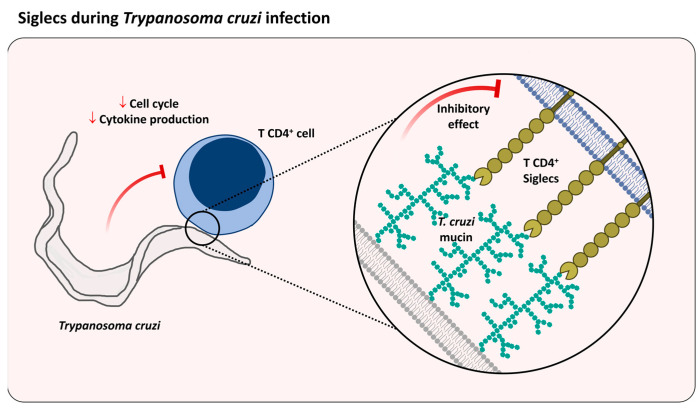
Differential roles of siglecs during *Trypanosoma cruzi* immunomodulation. Small arrows in red indicate downregulation and inhibitory effect is represented by red curved arrows.

In 2014, Jandus and colleagues demonstrated that siglec 7 exerts an essential function in tumor escape by disrupting the functions of natural killer cells [129]. Siglec 3, also known as CD33, is highly expressed on malignant blast cells and absent in normal hematopoietic pluripotent stem cells. It is suggested that CD33 expression may be involved in mechanisms related to drug resistance phenotype [129,130]. Siglec 15 is overexpressed in M2-Mɸ, and recognizes with high affinity the sialyl-Tn antigen. In this scenario, M2-Mɸ upregulates the production of TGF-β, which is known to be a cytokine with carcinogenic properties [131]. Other siglecs, such as siglec 9 and siglec 12 have been correlated with both tumor progression and immune evasion, since they were found to be overexpressed on different human epithelial carcinomas [132,133]. In 2013, we demonstrated that the inhibitory effects of *Trypanosoma cruzi* sialoglycoproteins on CD4^+^ T cells might be associated with increased susceptibility to infection. In this work, we suggest that the binding of sialoglycoproteins with siglecs would be involved in this process [134] (Figure 4). A variety of other siglec interactions with viral, protozoan pathogens and bacteria have been described elsewhere [135,136,137,138,139].

Thanks to advances in the field of glycoimmunology, today we know many siglec genes and binding specificities are quickly evolving among primates, with crucial extant polymorphisms in human populations that may impact vulnerability to infection-associated disorders [135]. Since carbohydrate–carbohydrate- and carbohydrate–protein-mediated interactions are essential for the maintenance of homeostasis [20], target pathways altered by such interactions are being identified as excellent therapeutic goals to combat different diseases, such as cancer [140,141]. It has become increasingly evident that the development of new therapeutic approaches is necessary to counter the long-term remission after cancer immunotherapy. In this line of thought, many research groups have focused their efforts on disturbing glycoimmune checkpoints, which may act as good targets for cancer treatment [142]. In this context, the most targeted pathways involve the vascular and immune circuits triggered by both galectins [143,144] and sialoglycan–siglec axis [145,146]. It is important to note that in both cases, there are already successful ongoing clinical trials [59,146,147].

## 5. Oncofetal Antigens as Modulators of the Immune Response

**Figure 5:**
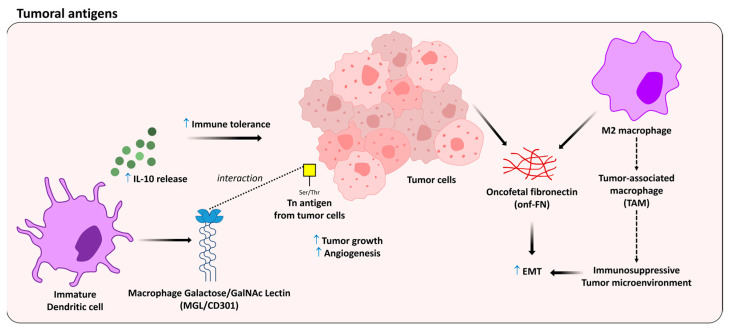
Immunomodulatory effects by tumoral antigens. Small arrows in blue indicate upregulation. Dashed line indicates receptor interaction and dashed arrows indicate successive effects.

Another topic that has grown exponentially in the field of glycoimmunology is the impact of oncofetal antigens on the immune system [148,149,150,151]. By definition, oncofetal proteins are generated in developing (fetal) as well as cancer (onco) cells. This expression can reproduce essential functions during development that are reactivated during cancer development and/or progression [152]. Usually, oncofetal proteins are decorated with truncated glycans, and many of these are used as glycobiomarkers for the diagnosis of different types of cancer [153]. Examples include the carcinoembryonic antigen [154], the prostate-specific antigen [155], and the CA-125 antigen [156], used as markers for colorectal, prostate, and ovarian cancers, respectively [157]. Usually, oncofetal proteins are able to elicit B cell-dependent immune responses (e.g., antibody production). It is important to note that self-antigens are not immunogenic, and therefore are not capable of inducing the and therefore are not capable of inducing the production of antibodies in an organism said as tolerant [158]. However, oncofetal epitopes are often immunogenic, since they are not widely expressed by adult health cells. In this context, the immune cells may elicit self-immunity under some conditions [159]. Among the unusual glycans carried by oncofetal proteins, Tn sialyl Tn antigens stand out [160,161]. Although many papers have demonstrated the importance of studying the immunobiological effects induced by the sialyl Tn antigen [162,163,164,165], in this last section of the article, we deal only with the Tn antigen, which is the simplest possible amino acid–carbohydrate glycoconjugate and comprises a 2-deoxy-2-acetamido-d-galactose (GalNAc) α-*O*-linked to either serine or threonine residues in a polypeptide chain [166]. In 1957, the Tn antigen was described by Moreau [167], and its structure was elucidated 18 years later [168]. The Tn antigen started to receive a lot of attention from 1974 onwards, when its high expression began to be observed in most tumors of epithelial origin, namely carcinomas and adenocarcinomas [169]. Nowadays, its high expression is known to be associated with a poor prognosis for different types of cancer [169], since it contributes to an immunosuppressive microenvironment and drives molecular pathways associated with metastasis [170]. Preexisting anticarcinoma anti-Tn antibodies are induced mainly by the intestinal flora and normally found in healthy individuals, while cellular immune responses to Tn epitopes are induced only by some lymphomas and carcinomas, in very early, including preclinical, cancer detection [171,172]. Tn antigen can be recognized by the Mɸ galactose/GalNAc lectin, known as MGL, which intermediates numerous immune tolerogenic and regulatory properties, mainly by reprogramming the maturation of dendritic cells [173]. Recently, da Costa and colleagues (2021) demonstrated that the Tn antigen induces the growth of lung tumors by promoting angiogenesis and immunosuppression through its interaction with MGL2 [174] (Figure 5).

Knowing that Tn antigen expression is practically absent in healthy tissue cells [162], in the early 1990s many synthetic carbohydrate vaccines began to be developed in order to determine their immunogenic potential [165,175]. Although chemically modified versions of the Tn antigen have shown better efficiency in some cases [175,176], there is still no vaccine based on the Tn antigen that is 100% effective and safe in controlling tumor progression in humans [177].

Over the past 10 years, our group has studied an atypical isoform of fibronectin (FN), called oncofetal FN (onf-FN), which was initially described by Hakomori in Seattle [178]. Onf-FN can be found in fetal tissues and tumor cells, and has been used for more than 25 years as a glycobiomarker [179,180,181]. This oncofetal isoform is characterized by an *O*-glycan linked to a specific threonine (Thr) residue, inside the type III homology connective segment (IIICS) domain of FN. The addition of a GalNAc unit to the Thr of the hexapeptide VTHPGY, generates a conformational change in the glycoprotein, creating a binding site for the FDC-6 mAb. Previous studies developed by Hakomori’s group showed that the Tn antigen carried by the glycoprotein acts as a minimal saccharide epitope to generate onf-FN [178,182], which in addition to being highly expressed by tumor cells and modulating the epithelial–mesenchymal transition (EMT) process [157,183,184,185,186], has also recently been detected in alternatively activated human macrophages [187], which display similar phenotypes of tumor-associated macrophages (TAMs) [188,189].

Recent works have demonstrated that Tn antigen expression promotes cancer metastasis through the activation of signaling pathways related to EMT [190,191]. These findings have important implications, since they strengthen the idea that aberrant glycosylation, especially the atypical expression of the Tn antigen, may be able to modulate the behavior of both tumor stromata and transformed cells. Further studies are necessary to better understand the real impact of truncated glycans, such as the Tn antigen, in oncobiology.

## 6. Conclusions

The data collected demonstrate a clear connection between glycobiology and immunology, with glycan epitopes playing a major role in the modulation of the immune system. In addition, the intense modulation dynamics of complex systems and the differential recognition of glycoconjugates also imply changes in recognition and establishment of an immune response. Atypical glycan structures also have great potential as immunotherapy tools for various diseases, and may in the future be used as a scaffold for biotechnological development in the treatment of numerous comorbidities. Therefore, continued investigation of this promising hot topic will establish important milestones for research in public health and technological development. As it stands, the importance of glycans in the onset, progression, and prognosis of several pathologies guarantees the marriage between immunology and glycobiology can never end in divorce.

## Data Availability

Not applicable.

## References

[1] Thomas D., Rathinavel A.K., Radhakrishnan P. (2021). Altered glycosylation in cancer: A promising target for biomarkers and therapeutics. Biochim. Biophys. Acta (BBA) Rev. Cancer.

[2] Go S., Yoshikawa M., Inokuchi J.-I. (2011). Glycoconjugates in the mammalian auditory system. J. Neurochem..

[3] Gabius H.-J., Manning J.C., Kopitz J., André S., Kaltner H. (2016). Sweet complementarity: The functional pairing of glycans with lectins. Cell. Mol. Life Sci..

[4] Chlubnová I., Sylla B., Nugier-Chauvin C., Daniellou R., Legentil L., Kralová B., Ferrières V. (2011). Natural glycans and glycoconjugates as immunomodulating agents. Nat. Prod. Rep..

[5] Esmail S., Manolson M.F. (2021). Advances in understanding N-glycosylation structure, function, and regulation in health and disease. Eur. J. Cell Biol..

[6] Sun X., Zhan M., Sun X., Liu W., Meng X. (2021). C1GALT1 in health and disease (Review). Oncol. Lett..

[7] Neuberger A. (1938). Carbohydrates in protein: The carbohydrate component of crystalline egg albumin. Biochem. J..

[8] Mendonça-Previato L., Todeschini A.R., Heise N., Previato J.O. (2005). Protozoan parasite-specific carbohydrate structures. Curr. Opin. Struct. Biol..

[9] Villena S.N., Pinheiro R.O., Pinheiro C.S., Nunes M.P., Takiya C.M., DosReis G.A., Previato J.O., Mendonça-Previato L., Freire-De-Lima C.G. (2008). Capsular polysaccharides galactoxylomannan and glucuronoxylomannan from *Cryptococcus neoformans* induce macrophage apoptosis mediated by Fas ligand. Cell. Microbiol..

[10] Dobrica M.-O., Lazar C., Branza-Nichita N. (2020). N-Glycosylation and N-Glycan Processing in HBV Biology and Pathogenesis. Cells.

[11] Limoli D.H., Jones C.J., Wozniak D.J. (2015). Bacterial Extracellular Polysaccharides in Biofilm Formation and Function. Microbiol. Spectr..

[12] Kumar A.S., Mody K., Jha B. (2007). Bacterial exopolysaccharides—A perception. J. Basic Microbiol..

[13] Spiro R.G. (2002). Protein glycosylation: Nature, distribution, enzymatic formation, and disease implications of glycopeptide bonds. Glycobiology.

[14] Wandall H.H., Nielsen M.A.I., King-Smith S., de Haan N., Bagdonaite I. (2021). Global functions of O-glycosylation: Promises and challenges in O-glycobiology. FEBS J..

[15] Nio-Kobayashi J. (2016). Tissue- and cell-specific localization of galectins, β-galactose-binding animal lectins, and their potential functions in health and disease. Anat. Sci. Int..

[16] Handa K., Hakomori S.-I. (2016). Changes of glycoconjugate expression profiles during early development. Glycoconj. J..

[17] Rudd P.M., Elliott T., Cresswell P., Wilson I.A., Dwek R.A. (2001). Glycosylation and the Immune System. Science.

[18] Arnold J.N., Dwek R.A., Rudd P.M., Sim R.B. (2006). Mannan binding lectin and its interaction with immunoglobulins in health and in disease. Immunol. Lett..

[19] Dos Reis J.S., Santos M.A.R.D.C., Mendonça D.P., Nascimento S.I.M.D., Barcelos P.M., de Lima R.G.C., da Costa K.M., Freire-De-Lima C.G., Morrot A., Previato J.O. (2022). Glycobiology of Cancer: Sugar Drives the Show. Medicines.

[20] Reily C., Stewart T.J., Renfrow M.B., Novak J. (2019). Glycosylation in health and disease. Nat. Rev. Nephrol..

[21] Schjoldager K.T., Narimatsu Y., Joshi H.J., Clausen H. (2020). Global view of human protein glycosylation pathways and functions. Nat. Rev. Mol. Cell Biol..

[22] Rabinovich G.A., Toscano M.A., Jackson S.S., Vasta G.R. (2007). Functions of cell surface galectin-glycoprotein lattices. Curr. Opin. Struct. Biol..

[23] Elola M.T., Blidner A.G., Ferragut F., Bracalente C., Rabinovich G.A. (2015). Assembly, organization and regulation of cell-surface receptors by lectin–glycan complexes. Biochem. J..

[24] Baum L.G., Cobb B.A. (2017). The direct and indirect effects of glycans on immune function. Glycobiology.

[25] Rabinovich G.A., Conejo-García J.R. (2016). Shaping the Immune Landscape in Cancer by Galectin-Driven Regulatory Pathways. J. Mol. Biol..

[26] An H.J., Froehlich J.W., Lebrilla C.B. (2009). Determination of glycosylation sites and site-specific heterogeneity in glycoproteins. Curr. Opin. Chem. Biol..

[27] Munkley J., Elliott D.J. (2016). Hallmarks of glycosylation in cancer. Oncotarget.

[28] Taniguchi N., Kizuka Y. (2015). Glycans and Cancer: Role of N-glycans in cancer biomarker, progression and metastasis, and therapeutics. Adv. Cancer Res..

[29] Nagae M., Kizuka Y., Mihara E., Kitago Y., Hanashima S., Ito Y., Takagi J., Taniguchi N., Yamaguchi Y. (2018). Structure and mechanism of cancer-associated N-acetylglucosaminyltransferase-V. Nat. Commun..

[30] Dhatchinamoorthy K., Colbert J.D., Rock K.L. (2021). Cancer Immune Evasion Through Loss of MHC Class I Antigen Presentation. Front. Immunol..

[31] Dong H., Strome S.E., Salomao D.R., Tamura H., Hirano F., Flies D.B., Roche P.C., Lu J., Zhu G., Tamada K. (2002). Tumor-associated B7-H1 promotes T-cell apoptosis: A potential mechanism of immune evasion. Nat. Med..

[32] Nardy A.F.F.R., Freire-De-Lima L., Freire-De-Lima C.G., Morrot A. (2016). The Sweet Side of Immune Evasion: Role of Glycans in the Mechanisms of Cancer Progression. Front. Oncol..

[33] Silva M.C., Fernandes A., Oliveira M., Resende C., Correia A., De-Freitas-Junior J.C.M., Lavelle A., Andrade-Da-Costa J., Leander M., Xavier-Ferreira H. (2020). Glycans as Immune Checkpoints: Removal of Branched N-glycans Enhances Immune Recognition Preventing Cancer Progression. Cancer Immunol. Res..

[34] Grigorian A., Demetriou M. (2011). *Mgat5* Deficiency in T Cells and Experimental Autoimmune Encephalomyelitis. ISRN Neurol..

[35] Lee S.-U., Grigorian A., Pawling J., Chen I.-J., Gao G., Mozaffar T., McKerlie C., Demetriou M. (2007). N-Glycan Processing Deficiency Promotes Spontaneous Inflammatory Demyelination and Neurodegeneration. J. Biol. Chem..

[36] Pereira M.S., Alves I., Vicente M., Campar A., Silva M.C., Padrão N., Pinto V., Fernandes A., Dias A., Pinho S.S. (2018). Glycans as Key Checkpoints of T Cell Activity and Function. Front. Immunol..

[37] Dias A.M., Correia A., Pereira M.S., Almeida C.R., Alves I., Pinto V., Catarino T.A., Mendes N., Leander M., Oliva-Teles M.T. (2018). Metabolic control of T cell immune response through glycans in inflammatory bowel disease. Proc. Natl. Acad. Sci. USA.

[38] Alves I., Fernandes A., Santos-Pereira B., Azevedo C.M., Pinho S.S. (2022). Glycans as a key factor in self and nonself discrimination: Impact on the breach of immune tolerance. FEBS Lett..

[39] De Bousser E., Meuris L., Callewaert N., Festjens N. (2020). Human T cell glycosylation and implications on immune therapy for cancer. Hum. Vaccines Immunother..

[40] Morgan R., Gao G., Pawling J., Dennis J.W., Demetriou M., Li B. (2004). *N*-Acetylglucosaminyltransferase V (Mgat5)-Mediated *N*-Glycosylation Negatively Regulates Th1 Cytokine Production by T Cells. J. Immunol..

[41] Ghosh C., Luong G., Sun Y. (2021). A snapshot of the PD-1/PD-L1 pathway. J. Cancer.

[42] Su C., Wang H., Liu Y., Guo Q., Zhang L., Li J., Zhou W., Yan Y., Zhou X., Zhang J. (2020). Adverse Effects of Anti-PD-1/PD-L1 Therapy in Non-small Cell Lung Cancer. Front. Oncol..

[43] Han Y., Liu D., Li L. (2020). PD-1/PD-L1 pathway: Current researches in cancer. Am. J. Cancer Res..

[44] Twomey J.D., Zhang B. (2021). Cancer Immunotherapy Update: FDA-Approved Checkpoint Inhibitors and Companion Diagnostics. AAPS J..

[45] Lei Q., Wang D., Sun K., Wang L., Zhang Y. (2020). Resistance Mechanisms of Anti-PD1/PDL1 Therapy in Solid Tumors. Front. Cell Dev. Biol..

[46] Cao P., Yang X., Liu D., Ye S., Yang W., Xie Z., Lei X. (2022). Research progress of PD-L1 non-glycosylation in cancer immunotherapy. Scand. J. Immunol..

[47] Wang Y.-N., Lee H.-H., Hsu J.L., Yu D., Hung M.-C. (2020). The impact of PD-L1 N-linked glycosylation on cancer therapy and clinical diagnosis. J. Biomed. Sci..

[48] Li C.-W., Lim S.-O., Xia W., Lee H.-H., Chan L.-C., Kuo C.-W., Khoo K.-H., Chang S.-S., Cha J.-H., Kim T. (2016). Glycosylation and stabilization of programmed death ligand-1 suppresses T-cell activity. Nat. Commun..

[49] Li C.-W., Lim S.-O., Chung E.M., Kim Y.-S., Park A.H., Yao J., Cha J.-H., Xia W., Chan L.-C., Kim T. (2018). Eradication of Triple-Negative Breast Cancer Cells by Targeting Glycosylated PD-L1. Cancer Cell.

[50] Lee H.-H., Wang Y.-N., Xia W., Chen C.-H., Rau K.-M., Ye L., Wei Y., Chou C.-K., Wang S.-C., Yan M. (2019). Removal of N-Linked Glycosylation Enhances PD-L1 Detection and Predicts Anti-PD-1/PD-L1 Therapeutic Efficacy. Cancer Cell.

[51] Liu K., Tan S., Jin W., Guan J., Wang Q., Sun H., Qi J., Yan J., Chai Y., Wang Z. (2020). N-glycosylation of PD-1 promotes binding of camrelizumab. EMBO Rep..

[52] Lu D., Xu Z., Zhang D., Jiang M., Liu K., He J., Ma D., Ma X., Tan S., Gao G.F. (2022). PD-1 N58-Glycosylation-Dependent Binding of Monoclonal Antibody Cemiplimab for Immune Checkpoint Therapy. Front. Immunol..

[53] Navarro P., Martínez-Bosch N., Blidner A.G., Rabinovich G.A. (2020). Impact of Galectins in Resistance to Anticancer Therapies. Clin. Cancer Res..

[54] Cagnoni A.J., Sáez J.M.P., Rabinovich G.A., Mariño K.V. (2016). Turning-Off Signaling by Siglecs, Selectins, and Galectins: Chemical Inhibition of Glycan-Dependent Interactions in Cancer. Front. Oncol..

[55] Jin F., Wang F. (2020). The physiological and pathological roles and applications of sialyl Lewis x, a common carbohydrate ligand of the three selectins. Glycoconj. J..

[56] Cerliani J.P., Blidner A.G., Toscano M.A., Croci D.O., Rabinovich G.A. (2017). Translating the ‘Sugar Code’ into Immune and Vascular Signaling Programs. Trends Biochem. Sci..

[57] Cummings R.D., Liu F.T., Rabinovich G.A., Stowell S.R., Vasta G.R., Varki A., Cummings R.D., Esko J.D., Stanley P., Hart G.W. (2022). Galectins. Essentials of Glycobiology.

[58] Compagno D., Jaworski F.M., Gentilini L., Contrufo G., Pérez I.G., Elola M.T., Pregi N., Rabinovich G.A., Laderach D.J. (2014). Galectins: Major Signaling Modulators Inside and Outside the Cell. Curr. Mol. Med..

[59] Stanczak M.A., Läubli H. (2022). Siglec receptors as new immune checkpoints in cancer. Mol. Asp. Med..

[60] Läubli H., Nalle S.C., Maslyar D. (2022). Targeting the Siglec–Sialic Acid Immune Axis in Cancer: Current and Future Approaches. Cancer Immunol. Res..

[61] Akimoto Y., Kawakami H. (2014). Histochemical Staining Using Lectin Probes. Methods Mol. Biol..

[62] Stoddart R.W., Jones C.J.P. (1998). Lectin Histochemistry and Cytochemistry-Light Microscopy: Avidin-Biotin Amplification on Resin- Embedded Sectlons. Methods Mol. Biol..

[63] Hashim O.H., Jayapalan J.J., Lee C.-S. (2017). Lectins: An effective tool for screening of potential cancer biomarkers. Peerj.

[64] Sato T. (2014). Lectin-Probed Western Blot Analysis. Methods Mol. Biol..

[65] Roth Z., Yehezkel G., Khalaila I. (2012). Identification and Quantification of Protein Glycosylation. Int. J. Carbohydr. Chem..

[66] Moriwaki K., Miyoshi E. (2014). Basic Procedures for Lectin Flow Cytometry. Methods Mol. Biol..

[67] Goumenou A., Delaunay N., Pichon V. (2021). Recent Advances in Lectin-Based Affinity Sorbents for Protein Glycosylation Studies. Front. Mol. Biosci..

[68] Pilobello K.T., Krishnamoorthy L., Slawek D., Mahal L.K. (2005). Development of a Lectin Microarray for the Rapid Analysis of Protein Glycopatterns. Chembiochem.

[69] Dang K., Zhang W., Jiang S., Lin X., Qian A. (2020). Application of Lectin Microarrays for Biomarker Discovery. Chemistryopen.

[70] Silva M.L.S. (2019). Lectin biosensors in cancer glycan biomarker detection. Adv. Clin. Chem..

[71] Yu H., Shu J., Li Z. (2020). Lectin microarrays for glycoproteomics: An overview of their use and potential. Expert Rev. Proteom..

[72] Trbojević-Akmačić I., Lageveen-Kammeijer G.S.M., Heijs B., Petrović T., Deriš H., Wuhrer M., Lauc G. (2022). High-Throughput Glycomic Methods. Chem. Rev..

[73] Syed P., Gidwani K., Kekki H., Leivo J., Pettersson K., Lamminmäki U. (2016). Role of lectin microarrays in cancer diagnosis. Proteomics.

[74] Puri K.D., Gopalakrishnan B., Surolia A. (1992). Carbohydrate binding specificity of the T_n_-antigen binding lectin from *Vicia villosa* seeds (VVLB_4_). FEBS Lett..

[75] Brooks S.A., Leathem A.J. (1995). Expression of alpha-GalNAc glycoproteins by breast cancers. Br. J. Cancer.

[76] Farrag F., Gewaily M., AbdElmaksoud A., Kassab M. (2017). Comparative glycoconjugates histochemistry of proventriculus of chicken, ducks and geese. Alex. J. Veter. Sci..

[77] Shibuya N., Goldstein I.J., Broekaert W.F., Nsimba-Lubaki M., Peeters B., Peumans W.J. (1987). The elderberry (*Sambucus nigra* L.) bark lectin recognizes the Neu5Ac(alpha 2-6)Gal/GalNAc sequence. J. Biol. Chem..

[78] Wu Z., Miller E., Agbandje-McKenna M., Samulski R.J. (2006). α2,3 and α2,6 N-Linked Sialic Acids Facilitate Efficient Binding and Transduction by Adeno-Associated Virus Types 1 and 6. J. Virol..

[79] Dodla M.C., Young A., Venable A., Hasneen K., Rao R.R., Machacek D.W., Stice S.L. (2011). Differing Lectin Binding Profiles among Human Embryonic Stem Cells and Derivatives Aid in the Isolation of Neural Progenitor Cells. PLoS ONE.

[80] Lu G., Holland L.A. (2018). Profiling the *N*-Glycan Composition of IgG with Lectins and Capillary Nanogel Electrophoresis. Anal. Chem..

[81] Kaneda Y., Whittier R.F., Yamanaka H., Carredano E., Gotoh M., Sota H., Hasegawa Y., Shinohara Y. (2002). The High Specificities of Phaseolus vulgaris Erythro- and Leukoagglutinating Lectins for Bisecting GlcNAc or β1–6-Linked Branch Structures, Respectively, Are Attributable to Loop B. J. Biol. Chem..

[82] Nishi N., Shoji H., Seki M., Itoh A., Miyanaka H., Yuube K., Hirashima M., Nakamura T. (2003). Galectin-8 modulates neutrophil function via interaction with integrin M. Glycobiology.

[83] Heyl K.A., Karsten C.M., Slevogt H. (2016). Galectin-3 binds highly galactosylated IgG1 and is crucial for the IgG1 complex mediated inhibition of C5aReceptor induced immune responses. Biochem. Biophys. Res. Commun..

[84] Giovannone N., Smith L.K., Treanor B., Dimitroff C.J. (2018). Galectin-Glycan Interactions as Regulators of B Cell Immunity. Front. Immunol..

[85] Vasta G.R., Quesenberry M., Ahmed H., O’Leary N. (1999). C-type lectins and galectins mediate innate and adaptive immune functions: Their roles in the complement activation pathway. Dev. Comp. Immunol..

[86] Baum L.G., Pang M., Perillo N.L., Wu T., Delegeane A., Uittenbogaart C.H., Fukuda M., Seilhamer J.J. (1995). Human thymic epithelial cells express an endogenous lectin, galectin-1, which binds to core 2 O-glycans on thymocytes and T lymphoblastoid cells. J. Exp. Med..

[87] Manzi M., Bacigalupo M.L., Carabias P., Elola M.T., Wolfenstein-Todel C., Rabinovich G.A., Espelt M.V., Troncoso M.F. (2015). Galectin-1 Controls the Proliferation and Migration of Liver Sinusoidal Endothelial Cells and Their Interaction with Hepatocarcinoma Cells. J. Cell. Physiol..

[88] Perillo N.L., Marcus M.E., Baum L.G. (1998). Galectins: Versatile modulators of cell adhesion, cell proliferation, and cell death. J. Mol. Med..

[89] Earl L.A., Bi S., Baum L.G. (2010). N- and O-Glycans Modulate Galectin-1 Binding, CD45 Signaling, and T Cell Death. J. Biol. Chem..

[90] Zuñiga E., Rabinovich G.A., Iglesias M.M., Gruppi A. (2001). Regulated expression of galectin-1 during B-cell activation and implications for T-cell apoptosis. J. Leukoc. Biol..

[91] Blidner A.G., Méndez-Huergo S.P., Cagnoni A.J., Rabinovich G.A. (2015). Re-wiring regulatory cell networks in immunity by galectin-glycan interactions. FEBS Lett..

[92] Thiemann S., Baum L.G. (2016). Galectins and Immune Responses—Just How Do They Do Those Things They Do?. Annu. Rev. Immunol..

[93] Toscano M.A., Bianco G.A., Ilarregui J.M., Croci D.O., Correale J., Hernandez J.D., Zwirner N., Poirier F., Riley E.M., Baum L.G. (2007). Differential glycosylation of TH1, TH2 and TH-17 effector cells selectively regulates susceptibility to cell death. Nat. Immunol..

[94] Rabinovich G.A., Ilarregui J.M. (2009). Conveying glycan information into T-cell homeostatic programs: A challenging role for galectin-1 in inflammatory and tumor microenvironments. Immunol. Rev..

[95] Sotomayor C.E., Rabinovich G.A. (2000). Galectin-1 Induces Central and Peripheral Cell Death: Implications in T-Cell Physiopathology. Dev. Immunol..

[96] Cooper D., Ilarregui J.M., Pesoa S.A., Croci D.O., Perretti M., Rabinovich G.A. (2010). Multiple Functional Targets of the Immunoregulatory Activity of Galectin-1: Control of immune cell trafficking, dendritic cell physiology, and T-cell fate. Methods Enzym..

[97] Croci D.O., Cerliani J.P., Dalotto-Moreno T., Méndez-Huergo S.P., Mascanfroni I.D., Dergan-Dylon S., Toscano M.A., Caramelo J.J., García-Vallejo J.J., Ouyang J. (2014). Glycosylation-Dependent Lectin-Receptor Interactions Preserve Angiogenesis in Anti-VEGF Refractory Tumors. Cell.

[98] Croci D.O., Cerliani J.P., Pinto N.A., Morosi L.G., Rabinovich G.A. (2014). Regulatory role of glycans in the control of hypoxia-driven angiogenesis and sensitivity to anti-angiogenic treatment. Glycobiology.

[99] Cagnoni A.J., Giribaldi M.L., Blidner A.G., Cutine A.M., Gatto S.G., Morales R.M., Salatino M., Abba M.C., Croci D.O., Mariño K.V. (2021). Galectin-1 fosters an immunosuppressive microenvironment in colorectal cancer by reprogramming CD8 ^+^ regulatory T cells. Proc. Natl. Acad. Sci. USA.

[100] Bacigalupo M.L., Manzi M., Espelt M.V., Gentilini L.D., Compagno D., Laderach D.J., Wolfenstein-Todel C., Rabinovich G.A., Troncoso M.F. (2015). Galectin-1 Triggers Epithelial-Mesenchymal Transition in Human Hepatocellular Carcinoma Cells. J. Cell. Physiol..

[101] You X., Wu J., Zhao X., Jiang X., Tao W., Chen Z., Huang C., Zheng T., Shen X. (2021). Fibroblastic galectin-1-fostered invasion and metastasis are mediated by TGF-β1-induced epithelial-mesenchymal transition in gastric cancer. Aging.

[102] Carabias P., Espelt M.V., Bacigalupo M.L., Rojas P., Sarrias L., Rubin A., Saffioti N.A., Elola M.T., Rossi J.P., Wolfenstein-Todel C. (2022). Galectin-1 confers resistance to doxorubicin in hepatocellular carcinoma cells through modulation of P-glycoprotein expression. Cell Death Dis..

[103] Strik H.M., Schmidt K., Lingor P., Tönges L., Kugler W., Nitsche M., Rabinovich G., Bähr M. (2007). Galectin-1 expression in human glioma cells: Modulation by ionizing radiation and effects on tumor cell proliferation and migration. Oncol. Rep..

[104] Wdowiak K., Francuz T., Gallego-Colon E., Ruiz-Agamez N., Kubeczko M., Grochoła I., Wojnar J. (2018). Galectin Targeted Therapy in Oncology: Current Knowledge and Perspectives. Int. J. Mol. Sci..

[105] Charkiewicz K., Goscik J., Raba G., Laudanski P. (2019). Syndecan 4, galectin 2, and death receptor 3 (DR3) as novel proteins in pathophysiology of preeclampsia. J Matern. Neonatal Med..

[106] Hepp P., Unverdorben L., Hutter S., Kuhn C., Ditsch N., Groß E., Mahner S., Jeschke U., Knabl J., Heidegger H.H. (2020). Placental Galectin-2 Expression in Gestational Diabetes: A Systematic, Histological Analysis. Int. J. Mol. Sci..

[107] Nachtigal M., Al-Assaad Z., Mayer E.P., Kim K., Monsigny M. (1998). Galectin-3 expression in human atherosclerotic lesions. Am. J. Pathol..

[108] Nachtigal M., Ghaffar A., Mayer E.P. (2008). Galectin-3 Gene Inactivation Reduces Atherosclerotic Lesions and Adventitial Inflammation in ApoE-Deficient Mice. Am. J. Pathol..

[109] MacKinnon A.C., Farnworth S.L., Hodkinson P.S., Henderson N.C., Atkinson K.M., Leffler H., Nilsson U.J., Haslett C., Forbes S.J., Sethi T. (2008). Regulation of Alternative Macrophage Activation by Galectin-3. J. Immunol..

[110] Li Y., Li T., Zhou Z., Xiao Y. (2022). Emerging roles of Galectin-3 in diabetes and diabetes complications: A snapshot. Rev. Endocr. Metab. Disord..

[111] Zangbede F.O.Q., Chauhan A., Sharma J., Mishra B.B. (2018). Galectin-3 in M2 Macrophages Plays a Protective Role in Resolution of Neuropathology in Brain Parasitic Infection by Regulating Neutrophil Turnover. J. Neurosci..

[112] Lv R., Bao Q., Li Y. (2017). Regulation of M1-type and M2-type macrophage polarization in RAW264.7 cells by Galectin-9. Mol. Med. Rep..

[113] Huflejt M., Leffler H. (2003). Galectin-4 in normal tissues and cancer. Glycoconj. J..

[114] Hokama A., Mizoguchi E., Sugimoto K., Shimomura Y., Tanaka Y., Yoshida M., Rietdijk S.T., de Jong Y.P., Snapper S.B., Terhorst C. (2004). Induced Reactivity of Intestinal CD4+ T Cells with an Epithelial Cell Lectin, Galectin-4, Contributes to Exacerbation of Intestinal Inflammation. Immunity.

[115] Mudter J., Neurath M.F. (2007). Il-6 signaling in inflammatory bowel disease: Pathophysiological role and clinical relevance. Inflamm. Bowel Dis..

[116] Barrès C., Blanc L., Bette-Bobillo P., André S., Mamoun R., Gabius H.-J., Vidal M. (2010). Galectin-5 is bound onto the surface of rat reticulocyte exosomes and modulates vesicle uptake by macrophages. Blood.

[117] Hong S.-H., Shin J.-S., Chung H., Park C.-G. (2019). Galectin-4 Interaction with CD14 Triggers the Differentiation of Monocytes into Macrophage-like Cells via the MAPK Signaling Pathway. Immune Netw..

[118] Bell S.L., Lopez K.L., Cox J.S., Patrick K.L., Watson R.O. (2021). Galectin-8 Senses Phagosomal Damage and Recruits Selective Autophagy Adapter TAX1BP1 To Control *Mycobacterium tuberculosis* Infection in Macrophages. Mbio.

[119] Bochner B.S., Zimmermann N. (2015). Role of siglecs and related glycan-binding proteins in immune responses and immunoregulation. J. Allergy Clin. Immunol..

[120] Murugesan G., Weigle B., Crocker P.R. (2021). Siglec and anti-Siglec therapies. Curr. Opin. Chem. Biol..

[121] Gonzalez-Gil A., Schnaar R.L. (2021). Siglec Ligands. Cells.

[122] Van Houtum E.J.H., Büll C., Cornelissen L.A.M., Adema G.J. (2021). Siglec Signaling in the Tumor Microenvironment. Front. Immunol..

[123] Smith B.A.H., Bertozzi C.R. (2021). The clinical impact of glycobiology: Targeting selectins, Siglecs and mammalian glycans. Nat. Rev. Drug Discov..

[124] Duan S., Paulson J.C. (2020). Siglecs as Immune Cell Checkpoints in Disease. Annu. Rev. Immunol..

[125] Crocker P.R. (2002). Siglecs: Sialic-acid-binding immunoglobulin-like lectins in cell–cell interactions and signalling. Curr. Opin. Struct. Biol..

[126] Angata T., von Gunten S., Schnaar R.L., Varki A., Varki A., Cummings R.D., Esko J.D., Stanley P., Hart G.W., Aebi M., Mohnen D., Kinoshita T., Packer N.H. (2022). I-Type Lectins. Essentials of Glycobiology.

[127] Perdicchio M., Ilarregui J.M., Verstege M.I., Cornelissen L.A.M., Schetters S.T.T., Engels S., Ambrosini M., Kalay H., Veninga H., Haan J.M.M.D. (2016). Sialic acid-modified antigens impose tolerance via inhibition of T-cell proliferation and de novo induction of regulatory T cells. Proc. Natl. Acad. Sci. USA.

[128] Merli M., Ferrario A., Maffioli M., Arcaini L., Passamonti F. (2015). Investigational therapies targeting lymphocyte antigens for the treatment of non-Hodgkin’s lymphoma. Expert Opin. Investig. Drugs.

[129] Jandus C., Boligan K.F., Chijioke O., Liu H., Dahlhaus M., Démoulins T., Schneider C., Wehrli M., Hunger R.E., Baerlocher G.M. (2014). Interactions between Siglec-7/9 receptors and ligands influence NK cell–dependent tumor immunosurveillance. J. Clin. Investig..

[130] Walter R.B., Gooley T.A., Van Der Velden V.H.J., Loken M.R., Van Dongen J.J.M., Flowers D.A., Bernstein I.D., Appelbaum F.R. (2007). CD33 expression and P-glycoprotein–mediated drug efflux inversely correlate and predict clinical outcome in patients with acute myeloid leukemia treated with gemtuzumab ozogamicin monotherapy. Blood.

[131] Takamiya R., Ohtsubo K., Takamatsu S., Taniguchi N., Angata T. (2012). The interaction between Siglec-15 and tumor-associated sialyl-Tn antigen enhances TGF- secretion from monocytes/macrophages through the DAP12-Syk pathway. Glycobiology.

[132] Mitra N., Banda K., Altheide T.K., Schaffer L., Johnson-Pais T.L., Beuten J., Leach R.J., Angata T., Varki N., Varki A. (2011). SIGLEC12, a Human-specific Segregating (Pseudo)gene, Encodes a Signaling Molecule Expressed in Prostate Carcinomas. J. Biol. Chem..

[133] Ibarlucea-Benitez I., Weitzenfeld P., Smith P., Ravetch J.V. (2021). Siglecs-7/9 function as inhibitory immune checkpoints in vivo and can be targeted to enhance therapeutic antitumor immunity. Proc. Natl. Acad. Sci. USA.

[134] Nunes M.P., Fortes B., Silva-Filho J.L., Terra-Granado E., Santos L., Conde L., Oliveira I.D.A., Freire-De-Lima L., Martins M.V., Pinheiro A.A.S. (2013). Inhibitory Effects of Trypanosoma cruzi Sialoglycoproteins on CD4+ T Cells Are Associated with Increased Susceptibility to Infection. PLoS ONE.

[135] Chang Y.-C., Nizet V. (2014). The interplay between Siglecs and sialylated pathogens. Glycobiology.

[136] Herzog S., Fragkou P.C., Arneth B.M., Mkhlof S., Skevaki C. (2022). Myeloid CD169/Siglec1: An immunoregulatory biomarker in viral disease. Front. Med..

[137] Mikulak J., Di Vito C., Zaghi E., Mavilio D. (2017). Host Immune Responses in HIV-1 Infection: The Emerging Pathogenic Role of Siglecs and Their Clinical Correlates. Front. Immunol..

[138] Chang Y.-C., Nizet V. (2020). Siglecs at the Host–Pathogen Interface. Adv. Exp. Med. Biol..

[139] Cavalcante T., Medeiros M.M., Mule S.N., Palmisano G., Stolf B.S. (2021). The Role of Sialic Acids in the Establishment of Infections by Pathogens, With Special Focus on Leishmania. Front. Cell. Infect. Microbiol..

[140] Stowell S.R., Ju T., Cummings R.D. (2015). Protein Glycosylation in Cancer. Annu. Rev. Pathol. Mech. Dis..

[141] Mereiter S., Balmaña M., Campos D., Gomes J., Reis C.A. (2019). Glycosylation in the Era of Cancer-Targeted Therapy: Where Are We Heading?. Cancer Cell.

[142] Bartish M., Del Rincón S.V., Rudd C.E., Saragovi H.U. (2020). Aiming for the Sweet Spot: Glyco-Immune Checkpoints and γδ T Cells in Targeted Immunotherapy. Front. Immunol..

[143] Videla-Richardson G.A., Morris-Hanon O., Torres N.I., Esquivel M.I., Vera M.B., Ripari L.B., Croci D.O., Sevlever G.E., Rabinovich G.A. (2021). Galectins as Emerging Glyco-Checkpoints and Therapeutic Targets in Glioblastoma. Int. J. Mol. Sci..

[144] Thijssen V.L., Rabinovich G.A., Griffioen A.W. (2013). Vascular galectins: Regulators of tumor progression and targets for cancer therapy. Cytokine Growth Factor Rev..

[145] Manni M., Läubli H. (2021). Targeting glyco-immune checkpoints for cancer therapy. Expert Opin. Biol. Ther..

[146] Bärenwaldt A., Läubli H. (2019). The sialoglycan-Siglec glyco-immune checkpoint—A target for improving innate and adaptive anti-cancer immunity. Expert Opin. Ther. Targets.

[147] Compagno D., Tiraboschi C., Garcia J.D., Rondón Y., Corapi E., Velazquez C., Laderach D.J. (2020). Galectins as Checkpoints of the Immune System in Cancers, Their Clinical Relevance, and Implication in Clinical Trials. Biomolecules.

[148] Sharma A., Seow J.J.W., Dutertre C.-A., Pai R., Blériot C., Mishra A., Wong R.M.M., Singh G.S.N., Sudhagar S., Khalilnezhad S. (2020). Onco-fetal Reprogramming of Endothelial Cells Drives Immunosuppressive Macrophages in Hepatocellular Carcinoma. Cell.

[149] Li D., Li N., Zhang Y.-F., Fu H., Feng M., Schneider D., Su L., Wu X., Zhou J., Mackay S. (2020). Persistent Polyfunctional Chimeric Antigen Receptor T Cells That Target Glypican 3 Eliminate Orthotopic Hepatocellular Carcinomas in Mice. Gastroenterology.

[150] Sun C., Lan P., Han Q., Huang M., Zhang Z., Xu G., Song J., Wang J., Wei H., Zhang J. (2018). Oncofetal gene SALL4 reactivation by hepatitis B virus counteracts miR-200c in PD-L1-induced T cell exhaustion. Nat. Commun..

[151] Elcheva I.A., Wood T., Chiarolanzio K., Chim B., Wong M., Singh V., Gowda C.P., Lu Q., Hafner M., Dovat S. (2020). RNA-binding protein IGF2BP1 maintains leukemia stem cell properties by regulating HOXB4, MYB, and ALDH1A1. Leukemia.

[152] Stern P.L., Schwab M. (2011). Oncofetal Antigen. Encyclopedia of Cancer.

[153] Buonaguro F.M., Pauza D., Tornesello M.L., Hainaut P., Franco R., Marincola F.M. (2014). Cancer Diagnostic and Predictive Biomarkers. BioMed Res. Int..

[154] Drake P.M., Cho W., Li B., Prakobphol A., Johansen E., Anderson N.L., Regnier F.E., Gibson B.W., Fisher S.J. (2010). Sweetening the Pot: Adding Glycosylation to the Biomarker Discovery Equation. Clin. Chem..

[155] Peracaula R., Tabarés G., Royle L., Harvey D.J., Dwek R.A., Rudd P.M., de Llorens R.R. (2003). Altered glycosylation pattern allows the distinction between prostate-specific antigen (PSA) from normal and tumor origins. Glycobiology.

[156] Saldova R., Struwe W.B., Wynne K., Elia G., Duffy M.J., Rudd P.M. (2013). Exploring the Glycosylation of Serum CA125. Int. J. Mol. Sci..

[157] Freire-De-Lima L. (2014). Sweet and sour: The impact of differential glycosylation in cancer cells undergoing epithelial-mesenchymal transition. Front. Oncol..

[158] Backes C., Ludwig N., Leidinger P., Harz C., Hoffmann J., Keller A., Meese E., Lenhof H.-P. (2011). Immunogenicity of autoantigens. BMC Genom..

[159] McClintock S.D., Warner R.L., Ali S., Chekuri A., Dame M.K., Attili D., Knibbs R.K., Aslam M.N., Sinkule J., Morgan A.C. (2015). Monoclonal antibodies specific for oncofetal antigen—Immature laminin receptor protein: Effects on tumor growth and spread in two murine models. Cancer Biol. Ther..

[160] Fu C., Zhao H., Wang Y., Cai H., Xiao Y., Zeng Y., Chen H. (2016). Tumor-associated antigens: Tn antigen, sTn antigen, and T antigen. Hla.

[161] Bulteau F., Thépaut M., Henry M., Hurbin A., Vanwonterghem L., Vivès C., Le Roy A., Ebel C., Renaudet O., Fieschi F. (2021). Targeting Tn-Antigen-Positive Human Tumors with a Recombinant Human Macrophage Galactose C-Type Lectin. Mol. Pharm..

[162] Loureiro L.R., Carrascal M.A., Barbas A., Ramalho J.S., Novo C., Delannoy P., Videira P.A. (2015). Challenges in Antibody Development against Tn and Sialyl-Tn Antigens. Biomolecules.

[163] Hakomori S.-I. (2001). Tumor-Associated Carbohydrate Antigens Defining Tumor Malignancy: Basis for Development of Anti-Cancer Vaccines. Adv. Exp. Med. Biol..

[164] Ibrahim N.K., Murray J.L. (2003). Clinical Development of the STn-KLH Vaccine (Theratope^®^). Clin. Breast Cancer.

[165] Julien S., Videira P.A., Delannoy P. (2012). Sialyl-Tn in Cancer: (How) Did We Miss the Target?. Biomolecules.

[166] Ju T., Otto V.I., Cummings R.D. (2011). The Tn Antigen-Structural Simplicity and Biological Complexity. Angew. Chem. Int. Ed..

[167] Moreau R., Dausset J., Bernard J., Moullec J. (1957). Acquired hemolytic anemia with polyagglutinability of erythrocytes by a new factor present in normal blood. Bull. Mem. La Soc. Med. Des Hop. Paris.

[168] Dahr W., Uhlenbruck G., Gunson H.H., Hart M. (1975). Molecular Basis of Tn-Polyagglutinability. Vox Sang..

[169] Ju T., Wang Y., Aryal R.P., Lehoux S.D., Ding X., Kudelka M.R., Cutler C., Zeng J., Wang J., Sun X. (2013). Tn and sialyl-Tn antigens, aberrant *O*-glycomics as human disease markers. Proteom. Clin. Appl..

[170] Cornelissen L.A.M., Blanas A., Zaal A., Van Der Horst J.C., Kruijssen L.J.W., O’Toole T., Van Kooyk Y., Van Vliet S.J. (2020). Tn Antigen Expression Contributes to an Immune Suppressive Microenvironment and Drives Tumor Growth in Colorectal Cancer. Front. Oncol..

[171] Springer G.F. (1997). Immunoreactive T and Tn epitopes in cancer diagnosis, prognosis, and immunotherapy. J. Mol. Med..

[172] Desai P.R. (2000). Immunoreactive T and Tn antigens in malignancy: Role in carcinoma diagnosis, prognosis, and immunotherapy. Transfus. Med. Rev..

[173] Da Costa V., Mariño K.V., Rodríguez-Zraquia S.A., Festari M.F., Lores P., Costa M., Landeira M., Rabinovich G.A., van Vliet S.J., Freire T. (2022). Lung Tumor Cells with Different Tn Antigen Expression Present Distinctive Immunomodulatory Properties. Int. J. Mol. Sci..

[174] Da Costa V., van Vliet S.J., Carasi P., Frigerio S., García P.A., Croci D.O., Festari M.F., Costa M., Landeira M., Rodríguez-Zraquia S.A. (2021). The Tn antigen promotes lung tumor growth by fostering immunosuppression and angiogenesis via interaction with Macrophage Galactose-type lectin 2 (MGL2). Cancer Lett..

[175] Toyokuni T., Hakomori S.-I., Singhal A.K. (1994). Synthetic carbohydrate vaccines: Synthesis and immunogenicity of Tn antigen conjugates. Bioorganic Med. Chem..

[176] Amedei A., Asadzadeh F., Papi F., Vannucchi M.G., Ferrucci V., Bermejo I.A., Fragai M., De Almeida C.V., Cerofolini L., Giuntini S. (2020). A Structurally Simple Vaccine Candidate Reduces Progression and Dissemination of Triple-Negative Breast Cancer. Iscience.

[177] Richichi B., Thomas B., Fiore M., Bosco R., Qureshi H., Nativi C., Renaudet O., BenMohamed L. (2014). A Cancer Therapeutic Vaccine based on Clustered Tn-Antigen Mimetics Induces Strong Antibody-Mediated Protective Immunity. Angew. Chem. Int. Ed..

[178] Matsuura H., Hakomori S. (1985). The oncofetal domain of fibronectin defined by monoclonal antibody FDC-6: Its presence in fibronectins from fetal and tumor tissues and its absence in those from normal adult tissues and plasma. Proc. Natl. Acad. Sci. USA.

[179] Loridon-Rosa B., Vielh P., Matsuura H., Clausen H., Cuadrado C., Burtin P. (1990). Distribution of oncofetal fibronectin in human mammary tumors: Immunofluorescence study on histological sections. Cancer Res.

[180] Kaczmarek J., Castellani P., Nicolo G., Spina B., Allemanni G., Zardi L. (1994). Distribution of oncofetal fibronectin isoforms in normal, hyperplastic and neoplastic human breast tissues. Int. J. Cancer.

[181] Mandel U., Therkildsen M.H., Reibel J., Sweeney B., Matsuura H., Hakomori S., Dabelsteen E., Clausen H. (1992). Cancer-associated changes in glycosylation of fibronectin. Immunohistological localization of oncofetal fibronectin defined by monoclonal antibodies. Apmis.

[182] Matsuura H., Takio K., Titani K., Greene T., Levery S.B., Salyan M.E., Hakomori S. (1988). The oncofetal structure of human fibronectin defined by monoclonal antibody FDC-6. Unique structural requirement for the antigenic specificity provided by a glycosylhexapeptide. J. Biol. Chem..

[183] Freire-De-Lima L., Gelfenbeyn K., Ding Y., Mandel U., Clausen H., Handa K., Hakomori S.-I. (2011). Involvement of O-glycosylation defining oncofetal fibronectin in epithelial-mesenchymal transition process. Proc. Natl. Acad. Sci. USA.

[184] Ding Y., Gelfenbeyn K., Freire-De-Lima L., Handa K., Hakomori S.-I. (2012). Induction of epithelial-mesenchymal transition with O-glycosylated oncofetal fibronectin. FEBS Lett..

[185] Alisson-Silva F., Freire-De-Lima L., Donadio J.L., Lucena M.C., Penha L., Sá-Diniz J.N., Dias W.B., Todeschini A.R. (2013). Increase of O-Glycosylated Oncofetal Fibronectin in High Glucose-Induced Epithelial-Mesenchymal Transition of Cultured Human Epithelial Cells. PLoS ONE.

[186] Da Fonseca L.M., da Silva V.A., da Costa K.M., dos Reis J.S., Previato J.O., Previato L.M., Freire-De-Lima L. (2022). Resistance to cisplatin in human lung adenocarcinoma cells: Effects on the glycophenotype and epithelial to mesenchymal transition markers. Glycoconj. J..

[187] Santos M.A.R.d.C., dos Reis J.S., Santos C.A.D.N., da Costa K.M., Barcelos P.M., Francisco K.Q.d.O., Barbosa P.A.G.N., da Silva E.D.S., Freire-De-Lima C.G., Morrot A. (2023). Expression of O-glycosylated oncofetal fibronectin in alternatively activated human macrophages. Immunol. Res..

[188] Wang H.-W., Joyce J.A. (2010). Alternative activation of tumor-associated macrophages by IL-4: Priming for protumoral functions. Cell Cycle.

[189] Aras S., Zaidi M.R. (2017). TAMeless traitors: Macrophages in cancer progression and metastasis. Br. J. Cancer.

[190] Liu Z., Liu J., Dong X., Hu X., Jiang Y., Li L., Du T., Yang L., Wen T., An G. (2019). Tn antigen promotes human colorectal cancer metastasis via H-Ras mediated epithelial-mesenchymal transition activation. J. Cell. Mol. Med..

[191] Dong X., Jiang Y., Liu J., Liu Z., Gao T., An G., Wen T. (2018). T-Synthase Deficiency Enhances Oncogenic Features in Human Colorectal Cancer Cells via Activation of Epithelial-Mesenchymal Transition. BioMed Res. Int..

